# Leveraging Scheme for Cross-Study Microbiome Machine Learning Prediction and Feature Evaluations

**DOI:** 10.3390/bioengineering10020231

**Published:** 2023-02-08

**Authors:** Kuncheng Song, Yi-Hui Zhou

**Affiliations:** 1 Bioinformatics Research Center, Biological Sciences, North Carolina State University, Raleigh, NC 27695, USA; 2 Departments of Biological Sciences and Statistics, North Carolina State University, Raleigh, NC 27695, USA

**Keywords:** feature selection, machine learning, microbiome, random forest, support vector machine, logistic regression

## Abstract

The microbiota has proved to be one of the critical factors for many diseases, and researchers have been using microbiome data for disease prediction. However, models trained on one independent microbiome study may not be easily applicable to other independent studies due to the high level of variability in microbiome data. In this study, we developed a method for improving the generalizability and interpretability of machine learning models for predicting three different diseases (colorectal cancer, Crohn’s disease, and immunotherapy response) using nine independent microbiome datasets. Our method involves combining a smaller dataset with a larger dataset, and we found that using at least 25% of the target samples in the source data resulted in improved model performance. We determined random forest as our top model and employed feature selection to identify common and important taxa for disease prediction across the different studies. Our results suggest that this leveraging scheme is a promising approach for improving the accuracy and interpretability of machine learning models for predicting diseases based on microbiome data.

## 1. Introduction

With the growth of our understanding of microbial fields and the advancement of sequencing technology, researchers across the globe have conducted various cohorts to investigate the relationship between microbes and host health [1]. The results from these studies have begun to unravel and confirm the critical role of microbiota in multiple health conditions, such as inflammatory bowel disease [2,3,4,5], colorectal cancer [6,7,8,9], and immune-related conditions, such as the response to immunotherapy [10,11,12,13,14]. 

One of the active fields from these studies focused on using compositional microbial data for machine learning methods to investigate the model prediction accuracy and determine which microbiota play a critical role in disease via feature selection [15,16] and differential abundance analyses [17,18,19,20]. Most of the studies focused on using a single dataset or multiple datasets from similar geographical locations to serve as the data for the training and testing of different machine learning models, which resulted in the overfitting of the machine learning models and reduced the generalizability of the results on external populations who share the same phenotypes. This lack of generalizability could contribute to different microbiota identification that could not be detected in another study with dissimilar demographics. In microbiome research, two crucial factors that have the potential to cause disparities in study results are the sample size and the microbial composition. The differences can influence the study design’s sample size, which can, in turn, affect the diversity of the microbial composition. For instance, studies with a larger sample size, encompassing a more extensive range of participants, tend to exhibit a more comprehensive range of microbial compositions, resulting in a higher number of unique operational taxonomic units (OTUs) or amplicon sequencing variants (ASVs) and thereby leading to more extensive taxonomic identifications at higher levels (such as the genus level). Conversely, studies with a smaller sample size are likely to yield fewer unique taxonomic identifications at all levels, potentially undermining machine learning algorithms’ accuracy and power. [21]. Moreover, microbial compositions can also vary due to factors such as diet, ethnicity, physical activity, age, medical history, and other characteristics. 

In our previous publication [16], we examined the impact of different machine learning methods using microbial data for binary classification, which identified tree-based methods such as random forest as the top performers, demonstrating consistency across different diseases. One of the most important features researchers perform with random forest models is to study the top features for microbial biomarker discovery [22,23,24]. However, the inter-study variation caused by the known disease-related taxa can rank differently from different datasets [25]. In addition, we noticed a drop in AUROC with cross-dataset analysis compared to within-study AUROC from previous studies using metagenomics data [24,25]. Here, three critical areas must be addressed to advance the cross-study investigation with microbiome data for disease prediction. Firstly, we need to understand the usefulness of 16S compared to metagenomics research since 16S is still the most cost-effective method compared to metagenomics sequencing with the potential for larger cohorts. Secondly, we need to develop a new method or approach that could retain the high model performance without the reduction present in the cross-study model. Lastly, we need to ensure the new method has the capability for microbial biomarker discovery of generalizable and consistent disease-related taxa.

In this study, we evaluated the efficacy of a leveraging scheme, wherein we constructed our predictive models by combining a portion of external data (target data) into a larger and independent data set (source data) for prediction and evaluation of the remaining portion of the external (target) data. The aim of this approach is to establish evidential relationships between microbial data from larger datasets, therefore enhancing the cross-study prediction performance and enabling the machine learning model to exhibit greater generalizability. Furthermore, this leveraging scheme approach offers a solution to the three previously identified problems. Similar leveraging schemes have been employed in the prediction of hospital records, demonstrating their viability [26,27], and this would be the first time records are evaluated using microbial data. We included nine different and independent 16S rRNA studies with three different diseases of interest: colorectal cancer (CRC), Crohn’s disease (CD), and immunotherapy response. We examined a number of factors that could affect the leveraging model prediction performances: 1. percentage of shared taxa between the source and target data (6 levels); 2. percentage of target samples in the source data (6 levels); 3. individual taxonomic levels and a stacked-taxa in which we combined data from phylum to species levels (7 levels). For each of these combinations, we used 100 iterations to draw different target samples for evaluation randomly, and each generated 468 unique training and testing dataset pairs for the evaluation of 13 machine learning methods. Our previous work confirmed good performance for random forest models on higher taxonomic levels, i.e., phylum [16]. For example, the lowest intra-study AUROC on the phylum level was around 0.75, which gradually increased to reach 0.875 at the genus level for the classification of Crohn’s disease. As a result, we can harness more information by stacking these taxonomic levels together to gain better model interpretability. In addition, our previous study also illustrated the importance of studying the taxa–taxa relationship from the cross-taxonomic level perspective, as individual species or groups of species under the same family could both play major roles in disease differentiation within machine learning models [28]. In the results, we showed this stacked-taxa dataset has competitive results to the genus level performance and can be used to identify generalizable and consistent taxa.

For the machine learning methods, we included support vector machine (SVM), which is a powerful but unstable machine learning method, as its performance tends to vary [16]. We also examined simple logistic regression and a few of its variations. Logistic regression with L2-regularization was evaluated to help improve the simple logistic regression with penalty terms, and we included two instance weights with L2 logistic regression that could help shift the source data distribution closer to the target data distribution using Euclidean [27,29] and Aitchison distances [30]. We also included k = 1, 2, and 3 weighted clusters, as suggested by Gong et al. [27]. The last groups of machine learning models are from the random forest, which has been shown to be one of the best and most consistent methods for microbial data prediction [16,25]. We examined the random forest classifier with case weights extracted from the two instance weights, which essentially gives samples closer to the target distribution higher chances of being drawn when generating each tree. Last but not least, we used feature selection methods for the random forest classifier to extract the core features and their feature importance information from both source and target data among our evaluated combinations for a better understanding of generalizable and consistent taxa.

Overall, we present a scheme for better understanding microbial features from different studies with the same disease of interest. We will also illustrate its application in feature selection for generalizable features across studies. 

## 2. Materials and Methods

### 2.1. Microbial Data Processing

We evaluated three diseases of interest, colorectal cancer (CRC), Crohn’s disease (CD), and immunotherapy responses. For both CRC and CD, we obtained two independent studies for each of these disease phenotypes. All of these datasets are 16S rRNA datasets collected from human fecal matter. The colorectal cancer datasets are from PRJNA290926 [6] and PRJEB6070 [7], and the Crohn’s disease datasets are from PRJEB13679 [5] and IBDMDB [2]. The preprocessing of the 16S rRNA was completed via QIIME2 (version 2021.4) with DADA2 algorithms using SILVA 138 reference database [31,32,33,34]. The finalized data were saved into phyloseq objects for easier downstream data manipulation. The immunotherapy data were downloaded in the format of phyloseq objects from GitHub published by Limeta et al. [35]; each study ASV and taxonomic table was extracted from the corresponding phyloseq objects to generate data frames similar to the CRC and CD data. Next, we removed samples with less than 1000 reads as these samples tend to have low-quality issues in terms of microbial diversity as well as sequencing-related issues [36]. We further filtered out any taxa with less than 10% prevalence across samples, representing rare taxonomic assignments, and the taxonomy information is illustrated in Table 1.

The differential abundance analysis with analysis of compositions of microbiomes with bias correction (ANCOM-BC) [37] was performed using the ANCOMBC R package on each of the six taxonomic levels independently, and the differential taxa were based on BH-adjusted *p*-values ≤ 0.05. The full ANCOM-BC results are in Supplementary Table S2. 

### 2.2. Evaluation Scheme

**Figure 1:**
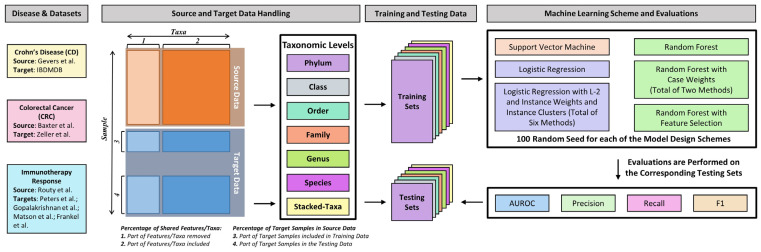
Leveraging scheme workflow.

The evaluation scheme is illustrated in Figure 1, which can be divided into four three sections. 

#### 2.2.1. Source and Target Data Handling

The source and target data for each of the comparisons were extracted from the corresponding phyloseq objects at each of the phylum, class, order, family, genus, and species levels, the raw count matrices were accumulated to form the individual taxonomic level count matrices and the stacked-taxa count matrices were created by stacking all the taxonomic levels matrices together. This step resulted in seven source-target paired datasets for each disease of interest comparison. 

#### 2.2.2. Training and Testing Data

When creating the training and testing data, we used a seed to randomly draw the designated proportion of the features/taxa between the source and target, followed by randomly selecting part of the target data to be added to the source data. This generates the training dataset. The remaining target data will be the testing dataset. Here, we are examining six different levels of shared features/taxa: 10%, 25%, 50%, 75%, 90%, and 100%, and we randomly selected target samples at 0%, 10%, 25%, 50%, 75%, and 90%. The 0% target samples in the source data serve as the baseline performance. We will use the newly generated training and testing to calculate the instance weights for each sample in the training set. We ran these combinations for 100 iterations, which gives 25,200 unique training and testing pairs for each disease of interest comparison. 

#### 2.2.3. Machine Learning Scheme and Evaluations

For each of the 25,200 training-testing pairs, we ran 13 machine-learning methods as detailed in Section 2.3, and for each of the machine-learning results, we extracted the Recall, Precision, F_1_, and AUROC metrics using the corresponding packages as detailed in Section 2.5. 

### 2.3. Machine Learning Models

#### 2.3.1. Logistic Regression 

Logistic regression is a common supervised machine learning method that can be used for the estimation of the log odds of the probability in a binary outcome based on the linear combination of the independent variables (features). While this method is popular, our previous investigation has shown that it is not a top-performing method for microbial data. In this study, we also explored adapted versions of logistic regression that incorporate sample weights. Specifically, we examined L2-regularized logistic regression with weighted instances and clusters, as described in [22]. The weighted instances were determined based on the Euclidean distance between each sample in the source dataset and the centroid of the target samples. In contrast, the weighted clusters were determined based on the k-nearest neighbors of 1, 2, or 3 clusters in the source dataset. The weights for the cluster method were based on the mean of the target data relative to the mean of each cluster in the source data. These methods aim to assign higher weights to source samples that are more similar to the target samples to improve model performance on testing data closer to the target dataset.

Additionally, we employed a modified version of the instance weights, which utilized the Aitchison distance [25] instead of the Euclidean distance. This modification was intended to better accommodate the compositional microbial data and improve the model’s fit. All of these approaches were implemented using the instance-weight function in LIBLINEAR [33] with L2-regularization and 5-fold cross-validation. For simplicity, we will refer to these models as L2 in this paper.

#### 2.3.2. Random Forest

Random forest is one of the most high-performing supervised learning methods in many fields. Essentially, random forest creates multiple decision trees (i.e., 500 trees in our analyses) on subsets of the training dataset, and the prediction of the decision is based on the combinatory power of these decision trees on the testing dataset. To better train the data, we also utilized the feature selection method from the Boruta package, and the random forest classifier is from the randomForest R package [38,39]. Essentially, the Boruta feature selection algorithm duplicates and randomly shuffles each row of each column to generate an artificial data frame and merges back with the original data. The random forest classifier will then run on the merged dataset, and the importance (Z-scores) for each of the real features will be evaluated and compared with its shuffled replicas, and if the original features performed better, it is considered a “Hit”. This will be run at multiple iterations, and the normalized hit will be extracted from these runs, representing the percentage of runs in which the features are considered important. In addition, we also used the Euclidean and Aitchison instance weights as the case weights (probability for the samples to be drawn during permutation) to evaluate the impact of the model using the ranger R package [40]. These case weights adjust the chance for the samples with higher values to be selected when generating the tree. Lastly, the “Normalized Hit” features are extracted from each of the 100 iterations, and an average normalized hit is calculated for each of the selected features/taxa. 

#### 2.3.3. Support Vector Machine

SVM is another popular supervised learning algorithm for classification problems. SVM aims to identify the optimal decision boundary known as the hyperplane in the high-dimensional that segregates the data points into classes. In this study, we focused on the linear SVM as our previous study has shown its high performance with microbial data for disease prediction [15].

### 2.4. Statistical Analysis

We used R (v4.1.0) [41] for data manipulation and statistical analyses for most of the analyses except for the logistic regression with L2-regularization performed using the LIBLINEAR [42] libraries in python (v3.7.10). The weight calculations were performed in R based on the methods from Gong et al. [27]. The alpha and beta diversities were calculated using vegan [43] and phyloseq [44] R packages. The Shannon index was used for alpha diversity with the pairwise Wilcoxon test for statistical difference evaluation. The Bray-Curtis dissimilarity [45] and permutational multivariate analysis of variance (PERMANOVA) [46] were used to analyze the beta-diversity when the sample size differences between the groups were less than 5-fold, and analysis of similarities (ANOSIM) [47] was performed otherwise. Both analyses were performed using 1000 permutations. Both diversities are performed on the genus-level after removing rare taxa that do not present in more than 10% of the samples. 

The model evaluation metrics were calculated in R with caret [48] and pROC [49] packages, and for LIBLINEAR models, we used the Scikit-learn [50] library for extracting the evaluation metrics. 

### 2.5. Evaluation Metrics

To evaluate the binary classification performance from the machine learning models, we focus on two parameters: F_1_ score and area under the receiver operating curve (AUROC) to give an overall performance of the models.

The F_1_ score (Equation (3)) is the summary of precision (Equation (1)) and recall (Equation (2)) using a harmonic mean. Precision represents the fraction of positive predictions which are correctly identified by the method (true positives) and recall details the fraction of positive cases which are correctly identified by the method. The F_1_ score provides an accurate measurement of the classification for both balanced and imbalanced datasets:

(1) $$\mathrm{precision}=\frac{\mathrm{True}\;\mathrm{Positive}}{\mathrm{True}\;\mathrm{Positive}+\mathrm{False}\;\mathrm{Positive}}$$

(2) $$\mathrm{recall}=\frac{\mathrm{True}\;\mathrm{Positive}}{\mathrm{True}\;\mathrm{Positive}+\mathrm{False}\;\mathrm{negative}}$$

(3) $$F_{1}=2\times \frac{\mathrm{precision}\times \mathrm{recall}}{\mathrm{precision}+\mathrm{recall}}$$

AUROC is used to examine the accurate binary classification of the outcome; the closer the AUROC to 1, the better the model’s ability to distinguish between the two classes, and an AUROC of 0.5 represents random guessing. 

### 2.6. Machine Learning Model Baseline 

To help evaluate the improvement or decline of the leveraging scheme, we use the 0% of the target samples in the source data as the baseline performance for the machine learning classifiers: SVM, simple logistic regression, logistic regression with L2-regularization, and all types of random forest models. For the logistic regression models with instance weights or weighted clusters, the logistic regression with L2-regularization at 0% target samples in the source data is used. 

## 3. Results

### 3.1. Microbial Differences among Study and Diagnosis

In this study, we examined three different phenotypes—colorectal cancer (CRC), Crohn’s disease (CD), and immunotherapy response—using nine independent datasets as described in Table 1. For each of these datasets, we compared disease cases to control patients, selecting the larger dataset as the source and the smaller dataset as the target. In general, the larger datasets contained a greater number of taxa, and our filtering procedures resulted in the removal of an average of 58.89%, 80.85%, and 37.50% of taxa for the CRC, CD, and immunotherapy comparisons, respectively. We focused on the shared taxa between the source and target datasets, which further reduced the number of taxa by 3% to 30%. Overall, we retained 354, 252, 437, 353, 413, and 428 taxa across six taxonomic levels for the CRC, CD, and four immunotherapy comparisons, respectively.

### 3.2. Microbial Inter and Intra-Diversity among Studies and Phenotypes

To understand the variations between the studies that shared the disease of interest, we evaluated the observed taxa variations between the studies and phenotypes using alpha (intra) and beta (inter) microbial diversities. Given larger datasets tend to generate more rare taxa, as illustrated by the comparison between Gevers et al. and IBDMBD data for Crohn’s disease, we will evaluate the alpha and beta diversities on filtered taxa after removing rare taxa that are not present in more than 10% of the samples. This filtering procedure reduces the complexity of the microbiome data while reserving the data integrity for better reproducibility and comparable data analysis [51].

**Figure 2:**
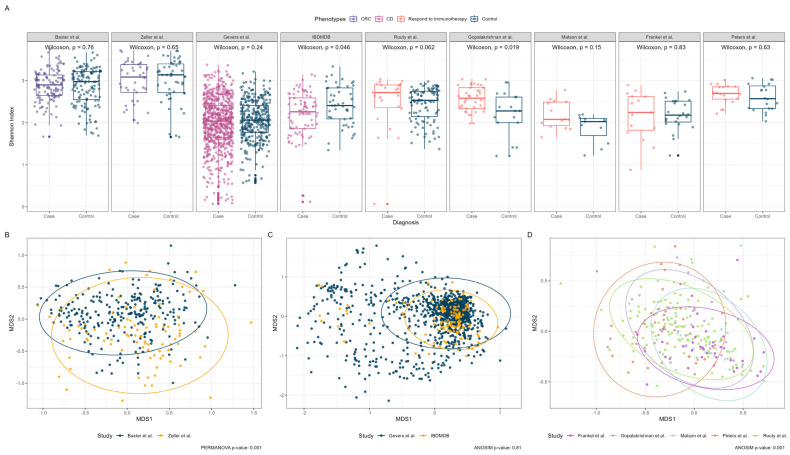
Alpha and beta diversity plots of the nine studies. (**A**) Alpha diversity boxplot on the post-filtering genus-level across nine studies between the Case and Control. Wilcoxon *p*-values are shown for each study. (**B**) Nonmetric multidimensional scaling plot of the Bray-Curtis dissimilarity matrix between two colorectal cancer studies. (**C**) Nonmetric multidimensional scaling plot of the Bray-Curtis dissimilarity matrix between two Crohn’s disease studies. (**D**) Nonmetric multidimensional scaling plot of the Bray-Curtis dissimilarity matrix among five immunotherapy studies. The PERMONAVA or ANOSIM *p*-values are shown for the beta-diversity plots. Note: For clear visualization, one sample from Routy et al. in panel D was removed from plotting due to its extremely high MDS1 values compared to all other samples.

As depicted in Figure 2A, the alpha diversity of most datasets at the genus level showed no significant differences, with the exception of the datasets from IBDMDB [2] and Gopalakrishnan et al. [11], which had Wilcoxon *p*-values of 0.046 and 0.019, respectively. In contrast, both the CRC (Figure 2B) and immunotherapy response (Figure 2D) datasets demonstrated significant differences between the studies at the genus level, with *p*-values less than 0.001. By contrast, the Crohn’s disease (Figure 2C) datasets showed no significant differences between the studies, with a *p*-value of 0.81.

### 3.3. Impact of Percentage of Shared Features/Taxa between Source and Target

**Figure 3:**
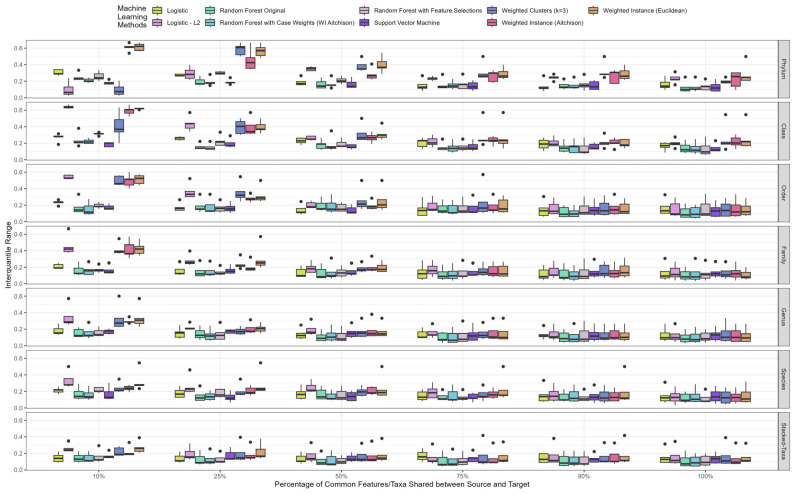
F_1_ score interquartile ranges for the percentage of common features/taxa shared between source and target across taxonomic levels and machine learning methods. The x-axis represents nine representative machine learning methods, and the y-axis represents the interquartile range calculated from 100 iterations of our random sampling scheme, and the solid black dot represents the outliers. The plot illustrated the results from colorectal cancer datasets.

In order to mimic real-world scenarios in which the number of taxa found can vary between studies, we analyzed the target and source using a range of percentages of shared features/taxa between them. We present the evaluation using the interquartile range (IQR) of the F_1_ score from colorectal cancer datasets across different combinations in Figure 3. It is ideal for these IQRs to be low and narrow, as this indicates consistent machine learning performance across our sampling schemes. We observed noticeable higher variations in most methods at 10% shared features/taxa between the target and source compared to higher percentages of shared features/taxa. Additionally, higher IQRs were more evident at higher taxonomic levels, such as the phylum and order levels. In general, we found that leveraging schemes that produce stable machine learning models contain at least 25–50% shared features between the target and source. In addition, the models are more consistent to the family, genus, species, and stacked-taxa levels, which are good candidates for our leveraging scheme evaluations.

### 3.4. Impact of Percentage of Target in Source Data Set

**Figure 4:**
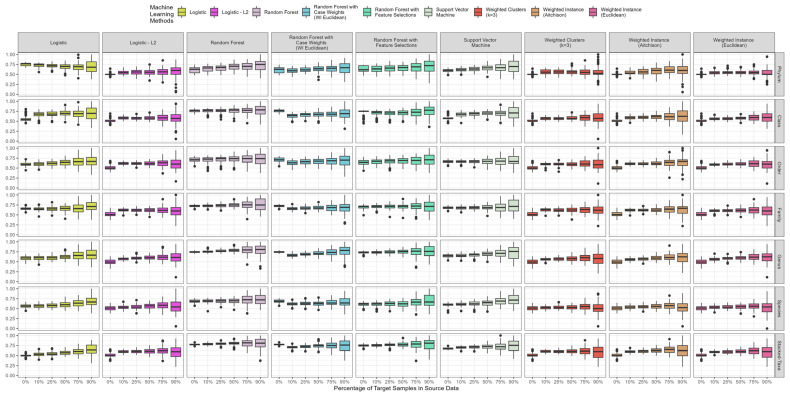
Area under the receiver operating curve for selected machine learning methods across different examined combinations. The x-axis represents the percentage of target samples combined into the source data to form the training data frame, which ranged from 0% (baseline, no leveraging) to 90%. The y-axis represents the area under the receiver operating curve, and the solid black dot represents the outliers. The plot illustrated the results from 75% of features/taxa shared between two colorectal cancer datasets.

To evaluate the impact of combining different percentages of target samples and their impact on the model performance, we average the AUROC across each of the 100 random sampling iterations. From Section 3.3, we confirm the minimal number of shared features/taxa for consistent results is 25%, and here we use 75% shared features/taxa combinations from our leveraging scheme. In Figure 4, we included the baseline results with 0% of the target sample in the source data. As the percentage of target samples in the source data increases, we observe a steady increase in the AUROC. However, at around 75% of target samples in the source data, the resulting AUROC becomes unstable, as indicated by the widened box-and-whisker ranges. This observation suggests that, when using our leveraging scheme, it is advisable to use at most 50% of the target samples in order to maintain stable results. The genus and stacked-taxa levels generate the highest AUROC compared to other methods, and the random forest with or without feature selections performed the best, followed by the SVM. The other methods did not perform as well as random forest and SVM.

### 3.5. Machine Learning Performance

Based on our results illustrated in Section 3.3 and Section 3.4, the evaluation of the machine learning models will use 25% and 50% percentage of target samples in the source data with 75% shared features/taxa between the target and source data. In Table 2, we compared the results of the leveraged models with their corresponding baseline models (i.e., 0% target samples in the source data), and we observed an increase in AUROC for most of the genus and stacked-taxa among the source-target comparisons. The random forest with or without feature selection is generally the top model, with SVM performing best for a few of the models. In general, by leveraging at least 50% of the target samples, the average model AUROC improved at most 0.042, 0.075, and 0.089 for the CRC, CD, and Immunotherapy data. All machine learning methods metrics summaries are in Supplementary Table S1. 

The logistic regression models with L2-Regularization performed poorly in comparison to the simple logistic regression models, as indicated by their AUROCs around 0.5 in Table 2. These models also tended to have convergence issues and failed to make predictions on the testing data, resulting in the assignment of all zeros to test samples. Similarly, the random forest model with case weights performed worse than the random forest models with or without feature selection.

Moreover, we could also observe the trends of performance across different taxonomic levels in Figure 4. With our best-performing methods, i.e., random forest with or without feature selection and support vector machine, the AUROC improves along with more detailed taxonomic information (i.e., phylum to stacked-taxa). However, we observed a drop in performance at the species level, which can be explained by three main factors. Firstly, 16S data do not have enough information to fully characterize the reads into species-level assignments, which directly relate to the sequencing length/quality, reference database completeness/correctness, etc. [52]. If the taxonomic assignment method cannot assign the reads to a species-level, it will cumulate the reads to a higher level (i.e., genus). Secondly, there are known misannotations of the species-level assignments (i.e., *Pseudomonas* species [53]), and some of the species undergo re-assignments with advanced research into a particular microbe (i.e., species under the genus *Bacillus* [54]). Lastly, as we have noticed in this study and previous study, more than 50% of the reads have missing assignments on the species-level, which negatively impact the machine learning performance [16]. All these factors diminish the quality of the species-level data, which reduces their corresponding performance. 

For the immunotherapy studies, the results have lower performance compared to CRC and CD, which is likely caused by the indirect relationship between the immunotherapy response and gut microbe. Four-fifths of these datasets had previously undergone meta-analysis using 3/5 of these immunotherapy studies (Gopalakrishnan et al., Matson et al., and Frankel et al.) as training and 1/5 (Peter et al.) as testing using random forest, which resulted in AUROC of 0.6. As shown in Table 2, our leveraging scheme was able to improve this for two of the four testing datasets in our design and enabled us to study generalizable taxa, as illustrated later. Even with the low model performance, the generalizable and consistent taxa investigation allows us to identify critical taxa that played major roles in immunotherapy responses (Section 2.6). 

### 3.6. Random Forest Top Predictors 

We further examined our best model random forest with feature selection methods to gain more biological relevance of the results. Here, we focused on the models with 100% features/taxa shared between the target and source. The top features were defined as the highest average normalized hits across 100 iterations across the different percentages of target samples in the source from 0% to 90% from both the individual taxonomic levels and stacked-taxa levels extracted from random forest models. The high importance represents more distinctive contributions to enable the random forest model to distinguish between the binary outcomes. 

**Figure 5:**
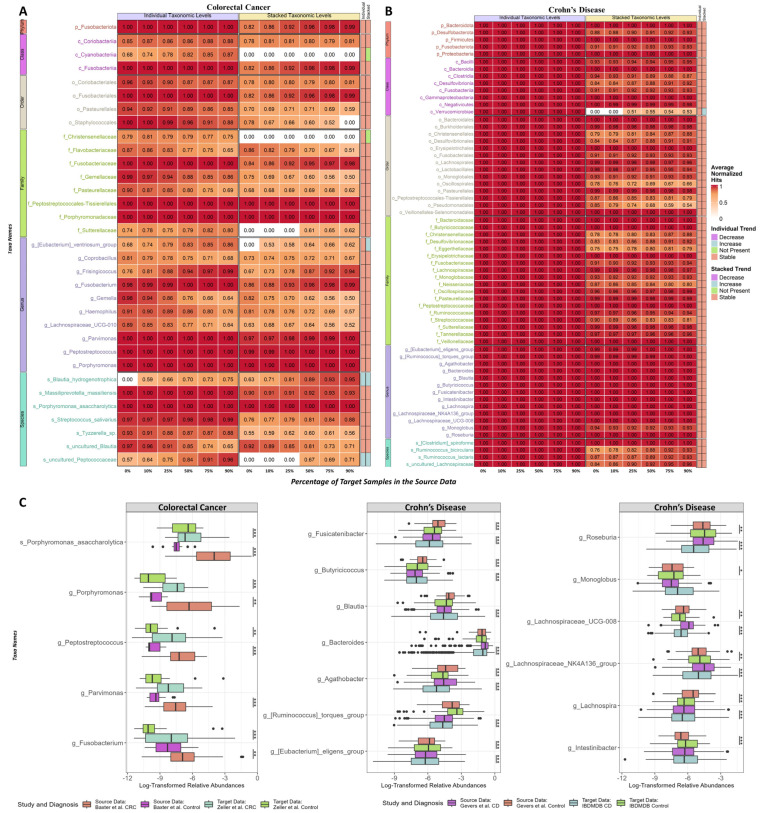
The top features of the random forest feature selection results for colorectal cancer and Crohn’s disease datasets were investigated for changes as the percentage of target samples in the source data increased. (**A**) presents a heatmap of the changes in the top 33 features for the colorectal cancer datasets at the individual taxonomic and stacked-taxa levels, while (**B**) shows the changes in the top 64 features for the Crohn’s disease datasets. (**C**) displays the log-transformed relative abundances for the consistent taxa identified between the individual taxonomic and stacked-taxa levels for the colorectal cancer and Crohn’s disease datasets. The solid black dots represent outliers. Taxa with a change of less than 0.15 between the baseline and importance with 50% of target samples in the source data were labeled as stable. Changes greater than 0.15 or less than −0.15 were labeled as increases or decreases, respectively. Taxa that were not selected as important by any of the models were labeled as “Not Present”. The *p*-value significance levels for taxa labeled as differentially abundant by ANCOM-BC are also shown. * indicates *p* < 0.05; ** indicates *p* < 0.001; *** indicates *p* < 0.0001.

Firstly, we observed high consistency between the individual taxonomic and the stacked-taxa between the source and target across all comparisons; the CRC and CD are shown in Figure 5A,B, respectively. By analyzing a larger number of target samples in the source data, we noticed that the importance of certain features tended to stay the same, while others either increased or decreased in importance. If the importance of certain taxa remains stable, this may suggest that they are generalizable features that are applicable across both the source and target datasets. On the other hand, an increase or decrease in importance may indicate that the taxa in question play different functional roles in the two studies. To make the interpretation of these findings easier, we defined an increase or decrease as a change of more than 0.15 in the average normalized hit between the baseline and 50% target samples in the source data. From Figure 5A,B, we can see that the stacked-taxa version has slightly lower average normalized hits due to the larger number of features/taxa in the data, but the overall trends are similar between the individual taxonomic and stacked-taxa versions. This supports the use of the stacked-taxa version for improved machine learning model interpretability while maintaining similar performance. Additionally, the results from the CRC and CD suggest that the leveraged random forest can consistently select stable features with at least 25% of the target samples in the source data.

For CRC, as shown in Figure 5A, the genus-level taxa *Fusobacterium* [55,56], *Pavimonas* [57,58], *Peptostreptococcus* [59], and *Porphyromonas* [60] (and *Porphyromonas asaccharolytica* [61]) are stable for both individual taxonomic and stacked-taxa levels models, and these are known to be associated with colorectal cancer. In Figure 5B, the importance for all individual taxonomic levels is rounded up to 1.00, indicating minimal impact with adding the target samples, and this is likely due to the much smaller target sample size compared to the source. The importance trends are more obvious in the stacked-taxa results. We focus on the genus-level taxa, which did not greatly lose more than 0.15 of their importance with the addition of target samples. These consistent taxa include many genus taxa that have documented connections with inflammatory bowel disease: the *Eubacterium eligens group* [62], the *Ruminoccocus torques group* [63], *Agathobacter* [64], *Bacteroides* [63,65,66], *Blautia* [63,65], *Butyricicoccus* [62], *Fusicatenibacter* [65], *Intestinibacter* [67], *Lachnospira* [66], *the Lachnospiraceae_NK4A136 group*, *Lachnospiraceae_UCG-008*, *Monoglobus* [68], and *Roseburia* [63,64,65,66]. The log-transformed relative abundances of these taxa from CRC and CD were illustrated in Figure 5C, and most of these consistent taxa were differentially abundant between the corresponding disease and control. Statistically significant differential taxa in both source and target are important for biomarker discovery for generalizable markers across studies. The ANCOM-BC results shown in Figure 5C identified many of these top-ranked taxa were differentially abundant between the case and controls from source or target datasets. 

**Figure 6:**
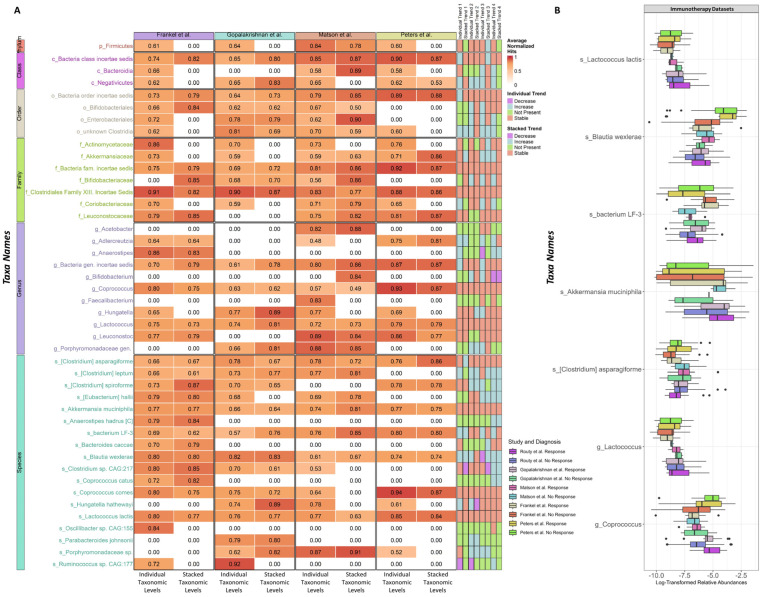
Top feature importance investigation changes for immunotherapy response datasets. (**A**) Heatmap for the changes in the top 44 features from the random forest feature selections result from individual taxonomic and stacked-taxa levels for colorectal cancer datasets across 50% of target samples in the source data. (**B**) Log-transformed relative abundances for the consistent taxa identified between the individual taxonomic and stacked-taxa for immunotherapy response. The solid black dots represent outliers. If there is no more than 0.15 change between the baseline and importance with 50% of target samples in the source for a taxon, it will be labeled as stable for the trend. For changes greater than 0.15 or less than −0.15, the trend is labeled as increase or decrease, respectively. If the features were not selected as important from any of the models, these taxa are labeled as “Not Present”. The trend categories are divided into four groups corresponding to different target data: 1. Peters et al.; 2. Gopalakrishnan et al.; 3. Matson et al.; 4: Frankel et al.

In contrast to the comparisons between the individual taxonomic and stacked-taxa levels for colorectal cancer and Crohn’s disease datasets, the immunotherapy comparisons showed reduced consistency. This is largely due to the low performance of the models that were trained using only microbiome data for immunotherapy response prediction. Nevertheless, we were still able to identify stable taxa that consistently served as good predictors for immunotherapy responses across all studies, as shown in Figure 6A. On the genus level, we observed two taxa *Coprococcus* [11] and *Lactococcus* [69], which have previously been identified as taxa with associations with immunotherapy. On the species level, our method was able to identify immunotherapy-related identities: *Clostridium asparagiforme* [70], *Akkermansia mucinphila* [70,71,72], *Bacterium LF-3* [73], *Blautia wexlerae* [71], and *Lactococcus lactis* [69]. Moreover, we also utilized a taxon from the class level to the genus level, referred to as an *incertae sedis*, in our random forest classifier. This decision was made due to the observed high significance of this unknown bacteria in the immunotherapy samples, which suggests there might be another group of microbes that play a significant role in immunotherapy that have yet to be identified. In addition, ANCOM-BC did not identify any genus and species-level taxa as being differentially abundant. This lack of detection could be due to the inconsistent performance of differential abundance analysis on microbial data, as noted in previous research [65]. Despite this limitation, our approach of leveraging diverse taxa offers a unique method for studying critical taxa that can be applied across multiple independent datasets. Figure 6B illustrates the log-transformed relative abundances of a set of taxa across the respective samples in five immunotherapy datasets. We observed that some taxa, such as genus *Coprococcus* and species *Bacterium LF-3*, *Akkermansia muciniphila*, and *Clostridium asparagiforme*, had observable different relative abundance range between the two outcomes group across all five studies. 

## 4. Conclusions

In this work, we presented a strategy for using microbial compositional data from one research to enhance the prediction of a second independent microbial dataset with the same illness of interest but a distinct population. We have shown that with 25% to 50% of the target samples in the source data, various machine-learning approaches may achieve enhanced prediction performance, with the random forest being the most effective. Next, we demonstrated that by using the feature selection methods in the random forest models from different leveraging schemes, we could interactively evaluate critical microbes at various taxonomic ranks to identify generalizable taxa that allow the random forest classifier to distinguish between the disease and control samples. As a result, these consistently high-importance taxa are good candidates for disease-related microbial biomarkers. Furthermore, we demonstrated the usefulness of the stacked-taxa data for machine learning models to concurrently analyze microorganisms from multiple taxonomic levels while retaining good model performance. Compared to previous work on cross-study investigations, our leveraging schemes offer a unique perspective, and we demonstrated its effectiveness and potential role in discovering generalizable disease-related taxa. 

Among all the taxonomic levels examined, the genus-level served as the best-performing level, and the species-level served as the most refined taxonomic level, albeit performing worse than the genus-level. While the performance might have dropped, this should not diminish the potentially useful information on the species-level, as many of these species are differentially abundant. As a solution, we recommend using the stacked taxa as the input for the leveraging scheme, which maintains a similar performance as the genus level and, more importantly, allows the researchers to simultaneously evaluate crucial taxa across all taxonomic levels with improved microbial interpretability of the results. 

This study has some limitations, including the potential for variability in the quality of the input data due to some samples being sequenced with shorter reads, which may affect the resolution and quality of the microbial taxonomic assignments. Additionally, the data on microbes alone may not provide a good prediction of immunotherapy response, and further studies using shotgun metagenomics and metabolomics may be needed to understand the role of various microbes more extensively in immunotherapy.

## Figures and Tables

**Table 1:** Study taxonomic summary.

Phenotypes	Study and Design Information	Number of Taxa Categories	Taxonomic Level						
			Phylum	Class	Order	Family	Genus	Species	Stacked-Taxa
Colorectal Cancer	Baxter et al. (Source) N = 261	Unique Taxa Count	19	32	73	135	373	387	1019
		Filtered Taxa Count	11	16	41	68	170	75	381
	Zeller et al. (Target) N = 91	Unique Taxa Count	22	39	91	159	400	384	1095
		Filtered Taxa Count	13	19	53	92	207	107	491
	Number of Shared Taxa between Target and Source Datasets for Colorectal Cancer Studies		11	16	40	66	156	65	354
Crohn’s Disease	Gevers et al. (Source) N = 1052	Unique Taxa Count	34	72	180	311	727	554	1878
		Filtered Taxa Count	9	12	34	53	117	34	259
	IBDMDB (Target) N = 128	Unique Taxa Count	35	70	162	247	489	315	1318
		Filtered Taxa Count	12	17	40	68	142	44	323
	Number of Shared Taxa between Target and Source Datasets for Crohn’s Disease Studies		9	12	34	53	113	31	252
Immunotherapy Responses	Routy et al. (Source) N = 127	Unique Taxa Count	14	31	49	83	191	595	963
		Filtered Taxa Count	12	23	33	55	110	251	484
	Peters et al. (Target) N = 27	Unique Taxa Count	13	26	41	69	155	409	713
		Filtered Taxa Count	12	24	35	60	110	261	502
	Number of Shared Taxa between Target and Source Datasets for Immunotheapy Response Studies		12	22	32	53	97	221	437
	Gopalakrishnan et al. (Target) N = 25	Unique Taxa Count	13	27	38	64	133	342	617
		Filtered Taxa Count	9	17	25	42	89	195	377
	Number of Shared Taxa between Target and Source Datasets for Immunotherapy Response Studies		9	17	25	40	82	180	353
	Matson et al. (Target) N = 39	Unique Taxa Count	13	24	39	68	154	482	780
		Filtered Taxa Count	12	22	34	60	113	261	502
	Number of Shared Taxa between Target and Source Dataset for Immunotherapy Response Studies		12	20	30	51	96	204	413
	Frankel et al. (Target) N = 39	Unique Taxa Count	14	28	43	75	175	533	868
		Filtered Taxa Count	11	23	36	60	129	317	576
	Number of Shared Taxa between Target and Source Dataset for Immunotherapy Response Studies		11	20	30	51	100	216	428

**Table 2:** Machine learning area under the curve (AUROC) for 25% and 50% target samples in source data. Gray-lighted cell contains the highest machine learning average AUROC for the taxonomic level and disease of interesting comparison set.

Disease of Interest		Taxonomic Levels	AUROC (AUROC Improvement from the Baseline Models)							
			Logistic	Logistic—L2	Logistic—L2 with Weighted Instance: Euclidean	Logistic—L2 with Weighted Clusters (k = 3)	Random Forest with Case Weights (WI: Euclidean)	Random Forest with Feature Selections	Random Forest	Support Vector Machine
50% of Target Samples in Source Data	Colorectal Cancer	Genus	0.621(+0.031)	0.605(+0.112)	0.602(+0.109)	0.576(+0.083)	0.712(+0.075)	0.753(+0.020)	0.789(+0.042)	0.697(+0.050)
		Stacked-Taxa	0.574(+0.077)	0.607(+0.096)	0.606(+0.095)	0.610(+0.098)	0.743(+0.055)	0.773(+0.022)	0.807(+0.031)	0.718(+0.043)
	Crohn’s Disease	Genus	0.756(+0.012)	0.515(+0.024)	0.545(+0.055)	0.554(+0.064)	0.783(+0.073)	0.842(+0.068)	0.841(+0.064)	0.784(+0.050)
		Stacked-Taxa	0.750(+0.034)	0.500(0.000)	0.567(+0.067)	0.500(0.000)	0.775(+0.086)	0.825(+0.075)	0.830(+0.074)	0.825(+0.043)
	Immunotherapy Dataset 1	Genus	0.601(+0.056)	0.500(+0.010)	0.499(+0.009)	0.500(+0.010)	0.503(+0.003)	0.683(+0.040)	0.649(+0.015)	0.591(+0.039)
		Stacked-Taxa	0.595(+0.043)	0.498(−0.005)	0.506(+0.003)	0.499(−0.004)	0.498(+0.003)	0.686(+0.103)	0.631(−0.017)	0.624(+0.104)
	Immunotherapy Dataset 2	Genus	0.534(+0.022)	0.500(−0.004)	0.500(−0.004)	0.497(−0.007)	0.500(+0.000)	0.564(+0.044)	0.651(+0.058)	0.615(+0.079)
		Stacked-Taxa	0.604(+0.025)	0.500(+0.006)	0.508(+0.015)	0.531(+0.037)	0.500(+0.000)	0.605(+0.066)	0.627(+0.089)	0.710(−0.007)
	Immunotherapy Dataset 3	Genus	0.581(+0.034)	0.500(+0.000)	0.501(+0.001)	0.507(+0.007)	0.528(+0.028)	0.581(+0.064)	0.594(+0.052)	0.567(+0.040)
		Stacked-Taxa	0.588(+0.030)	0.500(+0.005)	0.523(+0.028)	0.527(+0.032)	0.492(−0.003)	0.589(+0.041)	0.577(+0.066)	0.576(+0.007)
	Immunotherapy Dataset 4	Genus	0.580(+0.050)	0.497(−0.005)	0.488(−0.014)	0.490(−0.012)	0.476(−0.024)	0.606(+0.004)	0.600(−0.006)	0.579(+0.012)
		Stacked-Taxa	0.564(+0.026)	0.475(−0.024)	0.445(−0.054)	0.446(−0.053)	0.512(+0.018)	0.610(+0.014)	0.583(−0.009)	0.572(+0.010)
25% of Target Samples in Source Data	Colorectal Cancer	Genus	0.598(+0.007)	0.592(+0.099)	0.585(+0.092)	0.571(+0.078)	0.689(+0.052)	0.745(+0.011)	0.772(+0.025)	0.670(+0.023)
		Stacked-Taxa	0.548(+0.051)	0.604(+0.092)	0.592(+0.080)	0.604(+0.093)	0.723(+0.036)	0.765(+0.014)	0.795(+0.020)	0.709(+0.035)
	Crohn’s Disease	Genus	0.754(+0.010)	0.515(+0.024)	0.538(+0.048)	0.544(+0.054)	0.749(+0.039)	0.812(+0.038)	0.812(+0.035)	0.765(+0.030)
		Stacked-Taxa	0.745(+0.029)	0.500(0.000)	0.557(+0.057)	0.500(0.000)	0.737(+0.048)	0.791(+0.041)	0.798(+0.043)	0.810(+0.029)
	Immunotherapy Dataset 1	Genus	0.557(+0.012)	0.500(+0.010)	0.500(+0.010)	0.500(+0.010)	0.493(−0.006)	0.643(+0.000)	0.630(−0.003)	0.566(+0.013)
		Stacked-Taxa	0.572(+0.020)	0.500(−0.003)	0.498(−0.005)	0.496(−0.007)	0.502(+0.007)	0.630(+0.047)	0.622(−0.026)	0.568(+0.048)
	Immunotherapy Dataset 2	Genus	0.530(+0.017)	0.500(−0.004)	0.500(−0.004)	0.499(−0.005)	0.500(0.000)	0.568(+0.048)	0.626(+0.034)	0.570(+0.034)
		Stacked-Taxa	0.563(−0.016)	0.500(+0.006)	0.502(+0.008)	0.524(+0.030)	0.500(0.000)	0.567(+0.028)	0.594(+0.056)	0.693(−0.024)
	Immunotherapy Dataset 3	Genus	0.555(+0.008)	0.501(+0.001)	0.500(+0.000)	0.502(+0.002)	0.516(+0.016)	0.560(+0.043)	0.566(+0.025)	0.555(+0.028)
		Stacked-Taxa	0.571(+0.012)	0.500(+0.005)	0.506(+0.011)	0.519(+0.024)	0.505(+0.010)	0.554(+0.006)	0.548(+0.037)	0.547(−0.022)
	Immunotherapy Dataset 4	Genus	0.580(+0.050)	0.499(−0.003)	0.495(−0.007)	0.498(−0.004)	0.487(−0.013)	0.592(−0.010)	0.591(−0.015)	0.554(−0.013)
		Stacked-Taxa	0.548(+0.011)	0.490(−0.009)	0.473(−0.026)	0.474(−0.025)	0.504(+0.010)	0.577(−0.020)	0.586(−0.006)	0.552(−0.010)

## Data Availability

All microbial data were downloaded from public domain as detailed in Section 2.

## Supplementary Materials

The following supporting information can be downloaded at: https://www.mdpi.com/article/10.3390/bioengineering10020231/s1, Table S1: F_1_ and AUROC Summary Table; Table S2: ANCOM-BC Outputs.
